# Supplementary material for: Yeast Fin1-PP1 dephosphorylates an Ipl1 substrate, Ndc80, to remove Bub1-Bub3 checkpoint proteins from the kinetochore during anaphase
Source: PLoS Genet. 2021 May 25;17(5):e1009592. doi: 10.1371/journal.pgen.1009592 (PMC8184001; doi:10.1371/journal.pgen.1009592)
Supplement: S1 Table — (DOCX) [file pgen.1009592.s001.docx]

**S1 Table.** The list of yeast strains used in this study

| **Strains** | **Relevant genotypes** | **Reference** |
| --- | --- | --- |
| 2827-1-4 | MAT**a** *BUB1-GFP- Sphis5^+^ NUF2-mCherry* | Lab stock |
| 3196-1-3 | MAT**a** *fin1::KanMX BUB1-GFP::Sphis5⁺ NUF2-mCherry* | Lab stock |
| 3888-11-2 | MAT**a** *cdc15-2 BUB3-GFP::Sphis5⁺ NUF2-mCherry* | This study |
| 3890-14-3 | MAT**a** *cdc15-2 fin1::KanMX BUB3-GFP::Sphis5⁺ NUF2-mCherry* | This study |
| 4065-4-3 | MAT**a** *cdc15-2 fin1::KanMX bub1Δ::TRP1 BUB3-GFP::Sphis5⁺ NUF2-mCherry* | This study |
| 3177-3-4 | MAT**a** *cdc15-2 BUB1-GFP-Sphis5⁺ NUF2-mCherry* | Lab stock |
| 3196-3-1 | MAT**a** *cdc15-2 fin1::TRP1 BUB1-GFP-Sphis5⁺ NUF2-mCherry* | Lab stock |
| 3905-6-1 | MAT**a** *cdc15-2 fin1::TRP1 bub3::KanMX BUB1-GFP-Sphis5⁺ NUF2-mCherry* | This study |
| 3887-4-1 | MAT**a** *BUB1-GFP-Sphis5⁺ NUF2-mCherry* | This study |
| 3887-3-4 | MAT**α** *bub3::KanMX BUB1-GFP-Sphis5⁺ NUF2-mCherry* | This study |
| 4065-8-1 | MAT**α** *BUB3-GFP-Sphis5⁺ NUF2-mCherry* | This study |
| 4065-3-1 | MAT**α** *bub1::TRP1 BUB3-GFP-Sphis5⁺ NUF2-mCherry* | This study |
| 3175-1-4 | MAT**a** *NUF2-mCherry* | Lab stock |
| 3196-1-3 | MAT**a** *fin1::KanMX BUB1-GFP::Sphis5⁺ NUF2-mCherry* | Lab stock |
| 3777-2-1 | MAT**a** *fin1::KanMX PDS1-18myc-LEU2* | Lab stock |
| 2167-17-2 | MAT**a** *promURA3::tetR::GFP-LEU2 CENIV::tetOX448::URA3 TUB1-mCherry-URA3* | Lab stock |
| 3653-1-1 | MAT**a** *ipl1-321 fin1::TRP1 BUB1-GFP-Sphis5⁺ NUF2-mCherry* | This study |
| 3445-3-2 | MAT**a** *sli15-3 fin1::TRP1 BUB1-GFP-Sphis5⁺ NUF2-mCherry* | This study |
| 3922-11-3 | MAT**a** *fin1::TRP1 BUB3-GFP-Sphis5⁺ NUF2-mCherry* | This study |
| 3922-2-3 | MAT**a** *ipl1-321 fin1::TRP1 BUB3-GFP-Sphis5⁺ NUF2-mCherry* | This study |
| 3927-9-3 | MAT**a** *sli15-3 fin1::TRP1 BUB3-GFP-Sphis5⁺ NUF2-mCherry* | This study |
| 3802-1-4 | MAT**a** *NDC80-13myc-TRP1* | This study |
| 3802-2-2 | MAT**a** *fin1::KanMX NDC80-13myc-TRP1* | This study |
| 3893-10-2 | MAT**α** *NDC80::Sphis5⁺ NDC80-WT-myc-TRP1* | This study |
| 3897-4-4 | MAT**α** *NDC80::Sphis5⁺ NDC80-7A-myc-TRP1* | This study |
| 3893-10-3 | MAT**a** *fin1::KanMX NDC80::Sphis5⁺ NDC80-WT-myc-TRP1* | This study |
| 3897-5-3 | MAT**α** *fin1::KanMX NDC80::Sphis5⁺ NDC80-7A-myc-TRP1* | This study |
| 3975-5-3 | MAT**a** *cdc15-2 fin1::KanMX ndc80::Sphis5^+^ NDC80-WT-myc::TRP1 BUB1-GFP-Sphis5⁺ NUF2-mCherry* | This study |
| 3941-1-4 | MAT**a** *cdc15-2 fin1::KanMX NDC80::Sphis5⁺ NDC80-7A-myc-TRP1 BUB1-GFP-Sphis5⁺ NUF2-mCherry* | This study |
| 3893-9-2 | MAT**α** *cdc15-2 fin1::KanMX NDC80::Sphis5⁺ NDC80-WT-myc-TRP1 BUB3-GFP-Sphis5⁺ NUF2-mCherry* | This study |
| 4057-26-1 | MAT**α** *cdc15-2 fin1::KanMX NDC80::Sphis5⁺ NDC80-7A-myc-TRP1 BUB3-GFP-Sphis5⁺ NUF2-mCherry* | This study |
| SBY7258 | MAT**a** *NDC80::Sphis5^+^NDC80-WT-myc-TRP1* | Sue Biggins |
| SBY7259 | MAT**a** *NDC80::Sphis5^+^NDC80-7A-myc-TRP1* | Sue Biggins |
| 3932-5-1 | MAT**a** *NDC80::Sphis5⁺ NDC80-7A-myc-TRP1 PDS1-18myc-LEU2* | This study |
| 3932-3-1 | MAT**a** *fin1::KanMX NDC80::Sphis5⁺ NDC80-7A-myc-TRP1 PDS1-18myc-LEU2* | This study |
| 3460-1-2 | MAT**a** *cdc15-2* *MAD1-3GFP::Sphis5*⁺ *NUF2-mCherry* | This study |
| 3457-1-3 | MAT**a** *cdc15-2 fin1::KanMX MAD1-3GFP::Sphis5*⁺ *NUF2-mCherry* | This study |
| 3536-1-3 | MAT**a** *cdc14-2 fin1::KanMX* | This study |
| 4065-5-1 | MAT**α**  *fin1::KanMX BUB3-GFP-Sphis5^+^ NUF2-mCherry* | This study |
| 4159-6-4 | MAT**a** *TUB1-GFP-LEU* | This study |
| 4159-4-1 | MAT**a** *ctf13-30 TUB1-GFP-LEU2* | This study |
| 4146-2-4 | MAT**α** *mps1-1 BUB1-GFP- Sphis5^+^ NUF2-mCherry* | This study |
| 4171-3-1 | MAT**a** *mps1-1 fin1::KanMX BUB1-GFP-Sphis5^+^ NUF2-mCherry* | This study |
| DS002 | MAT**a** *cdc15-2 MPS1-13myc--Sphis5^+^* | This study |
| DS003 | MAT**a** *cdc15-2 fin1::TRP1 MPS1-13myc-Sphis5^+^* | This study |
| DS004 | MAT**a** *cdc15-2 MPS1-13myc- Sphis5^+^ DSN1-FLAG-URA3* | This study |
| DS005 | MAT**a** *cdc15-2 fin1::TRP1 MPS1-13myc- Sphis5^+^ DSN1-FLAG-URA3* | This study |
