# Supplementary material for: Yeast Fin1-PP1 dephosphorylates an Ipl1 substrate, Ndc80, to remove Bub1-Bub3 checkpoint proteins from the kinetochore during anaphase
Source: PLoS Genet. 2021 May 25;17(5):e1009592. doi: 10.1371/journal.pgen.1009592 (PMC8184001; doi:10.1371/journal.pgen.1009592)
Supplement: S2 Table — (DOCX) [file pgen.1009592.s002.docx]

|  |  |  |
| --- | --- | --- |

**S2 Table.** The list of plasmids used in this study

| **Names** | **Relevant genes** | **Reference** |
| --- | --- | --- |
| pRS416  pYW200  pMB6  pMB7  pSB1252  pSB1359 | *CEN-URA3* vector  *P_GAL_-myc-CIK1-CC*  *pFIN1-FIN1-TRP1 pFIN1-fin1-5A-TRP1*  *pFIN1-FIN1-GFP-LEU2 pFIN1-fin1-5A-GFP-LEU2* | Phil Hieter  Lab stock  This study  This study  Sue Biggins  Sue Biggins |
