# Supplementary figures and images for: Yeast Fin1-PP1 dephosphorylates an Ipl1 substrate, Ndc80, to remove Bub1-Bub3 checkpoint proteins from the kinetochore during anaphase
Source: PLoS Genet. 2021 May 25;17(5):e1009592. doi: 10.1371/journal.pgen.1009592 (PMC8184001; doi:10.1371/journal.pgen.1009592)

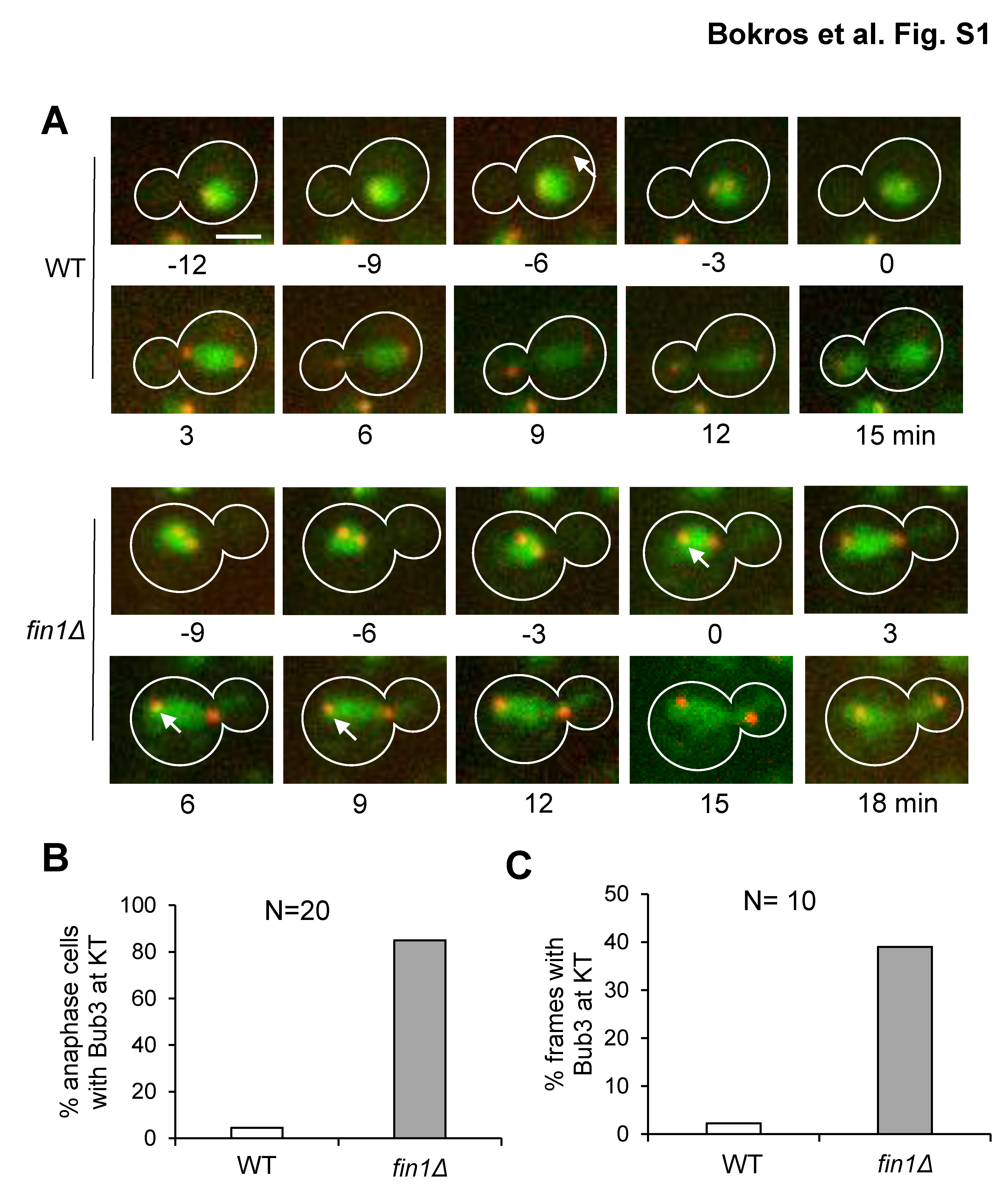

Supplement: S1 Fig — (A) Live-cell imaging of WT (4065-8-1) and fin1Δ (4065-5-1) cells to show kinetochore association of Bub3 in anaphase. Log-phase WT and fin1Δ cells with Bub3-GFP and Nuf2-mCherry were spotted onto an agarose pad filled with synthetic complete medium and subjected to live-cell microscopy. We set time 0 as the last point when the distance between two Nuf2-mCherry foci is less than 3μm. Arrows indicate co-localization of Bub3-GFP and Nuf2-mCherry. (B) Cells that had Bub3-GFP co-localization with Nuf2-mCherry at any time during anaphase was counted as positive and the percentage of positive cells was graphed (n = 20). KT: kinetochore. (C) The co-localization of Bub3-GFP with the kinetochore is dynamic after anaphase onset. The co-localization of Bub3 with either one or both kinetochore clusters during anaphase was counted for each frame of WT and fin1Δ cells during anaphase. We counted 10 cells for each strain. (TIF) [file pgen.1009592.s003.tif]

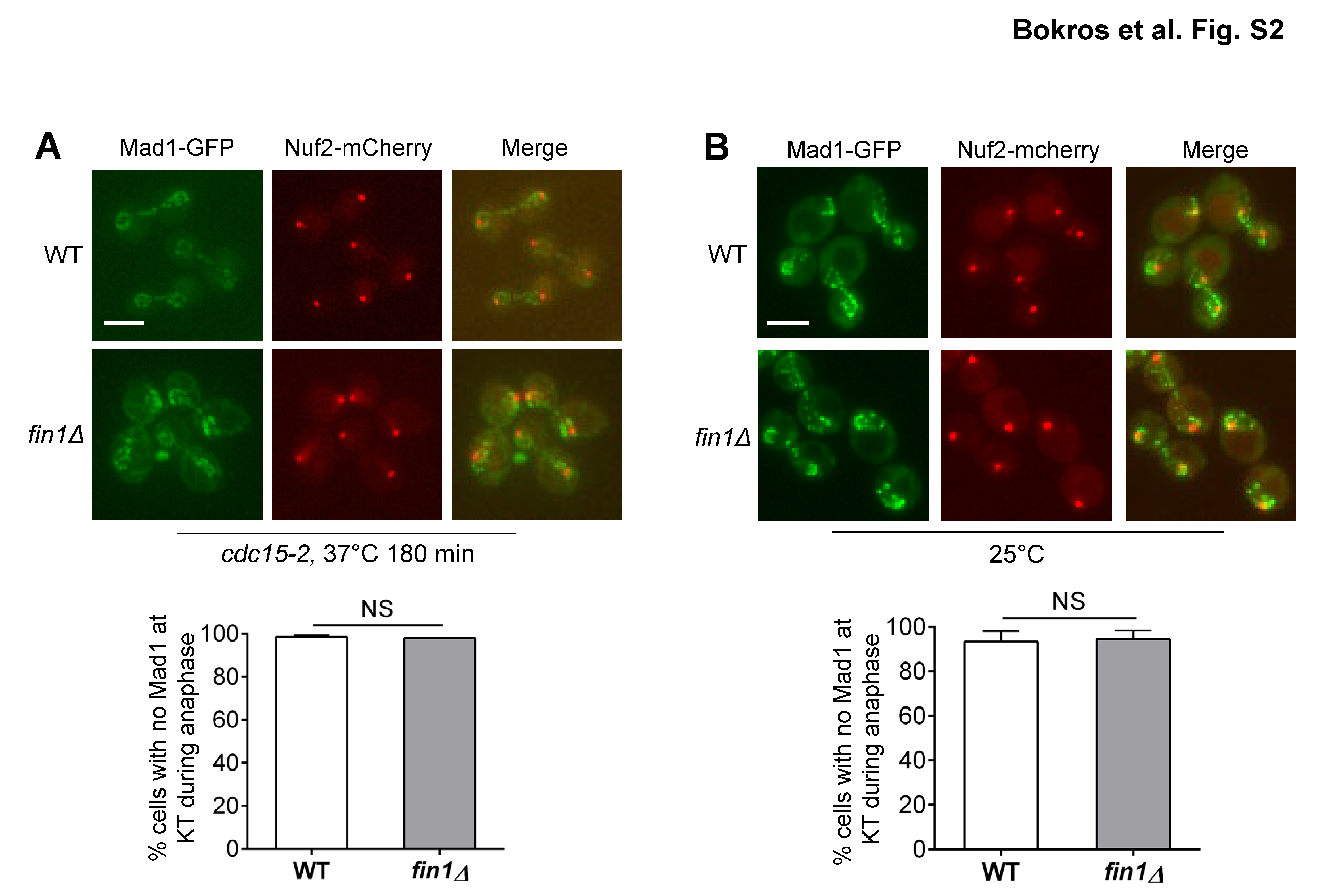

Supplement: S2 Fig — (A) cdc15-2 (3460-1-2) and cdc15-2 fin1Δ (3457-1-3) cells carrying Mad1-GFP and Nuf2-mCherry were grown to log phase at 25°C and then shifted to 37°C for 180 minutes. Samples were collected and pictures were taken. The frequency of cells without co-localization of Mad1-GFP and Nuf2-mCherry signals was counted for each strain (n = 100). The picture is representative of three experimental repeats and statistical significance determined by p < 0.05 using Wilcoxon rank sum test. Scale bar, 5 μm. (B) WT (3460-1-2) and fin1Δ (3457-1-3) cells containing Mad1-GFP and Nuf2-mCherry were grown to log phase at 25°C. Asynchronous cell samples were collected and pictures were taken. The frequency of co-localization of Mad1-GFP and Nuf2-mCherry signals during anaphase was counted for each strain (n = 100). Anaphase was determined when the distance between the two Nuf2 foci was greater than 3μM. The picture is a representative of three experimental repeats and statistical significance determined by p < 0.05 using Wilcoxon rank sum test. Scale bar, 5 μm. (TIF) [file pgen.1009592.s004.tif]

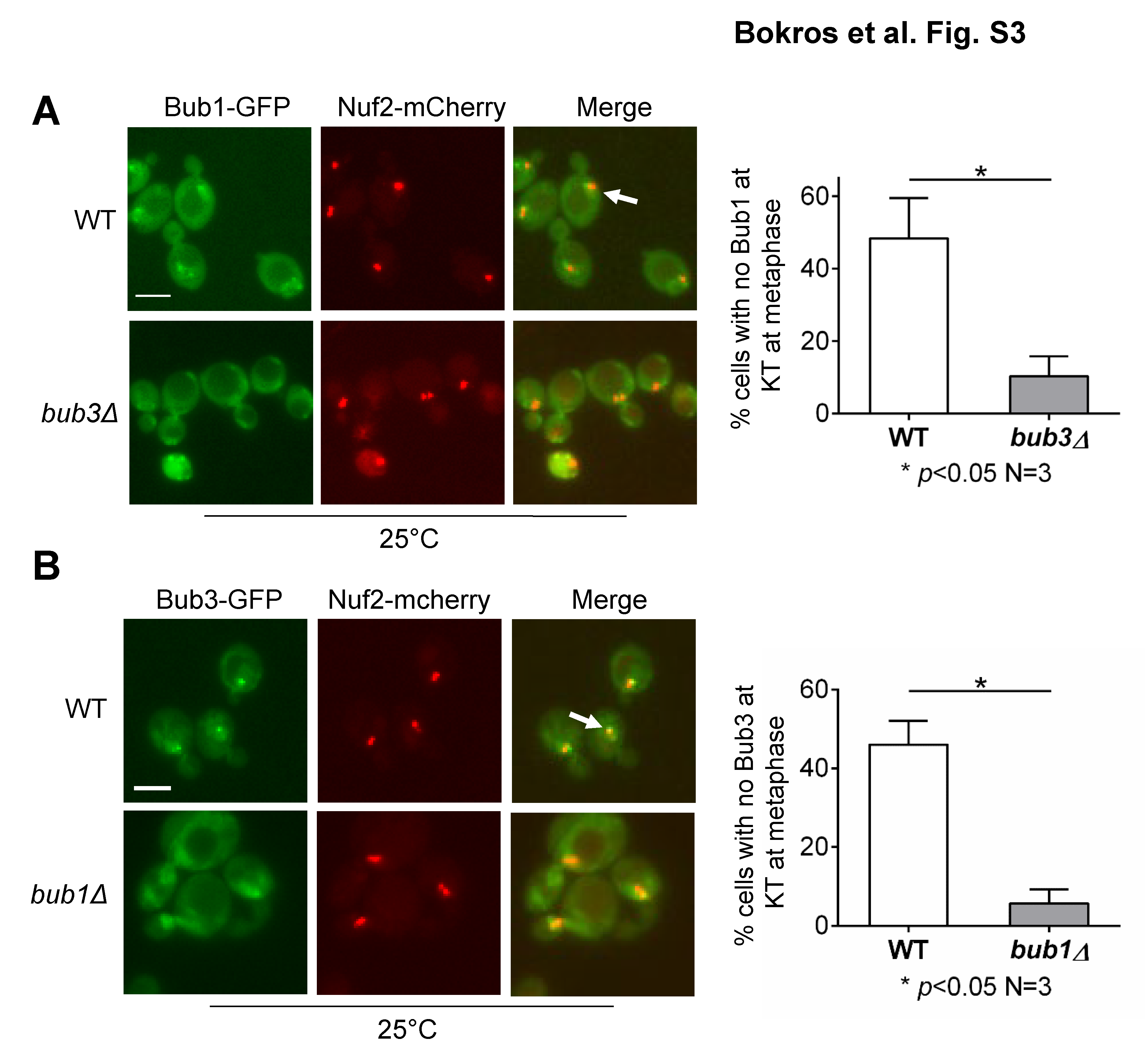

Supplement: S3 Fig — (A) WT (2827-1-4) and bub3Δ (3887-3-4) cells containing Bub1-GFP and Nuf2-mCherry were grown to log phase at 25°C. Asynchronous cells were collected and pictures were taken. The frequency of co-localization of Bub1-GFP and Nuf2-mCherry signals in metaphase was counted for each strain (n = 100). Metaphase cells are large-budded with two Nuf2 clusters less than 3μm apart. White arrows indicate Bub1-kinetochore co-localization. The experiment was repeated three times and statistical significance was determined by p < 0.05 using Wilcoxon rank sum test. Scale bar, 5 μm. (B) WT (4065-8-1) and bub1Δ (4065-3-1) cells containing Bub3-GFP and Nuf2-mCherry were grown as described above. The same method was used to compare the frequency of Bub3-Nuf2 co-localization. Scale bar, 5 μm. (TIF) [file pgen.1009592.s005.tif]

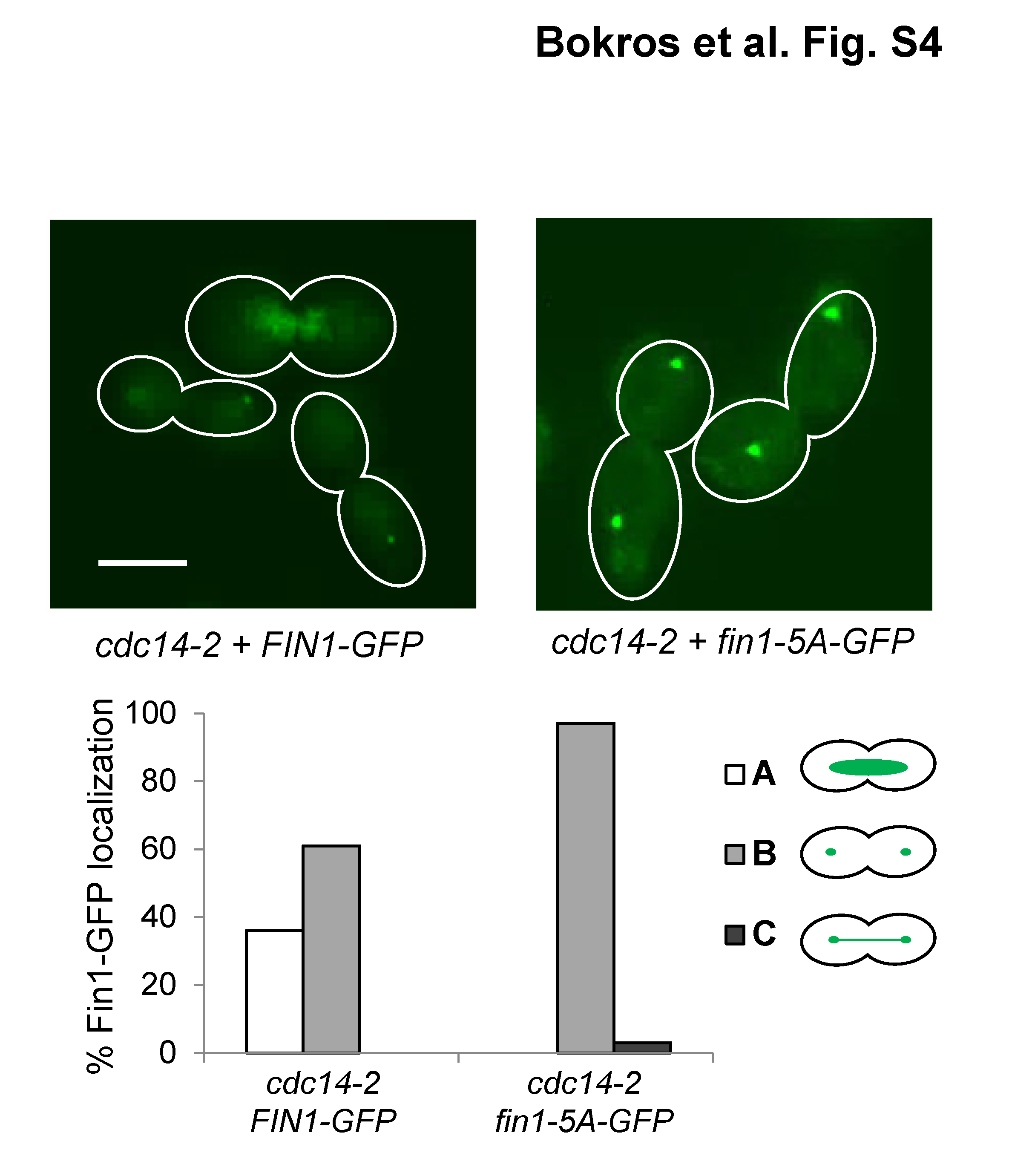

Supplement: S4 Fig — cdc14-2 (3536-1-2) cells with FIN1-GFP (pSB1252) or fin1-5A-GFP (pSB1359) plasmid were grown at 25°C until log-phase, and then transferred to 37°C for 180 minutes. Cells were collected and fixed to visualize GFP signal. Cells with different patterns of GFP signal were counted (n = 100). Representative images are shown on the top. Scale bar; 5μm. The percentage of cells with different patterns of GFP signals is shown in the bottom panel. (TIF) [file pgen.1009592.s006.tif]

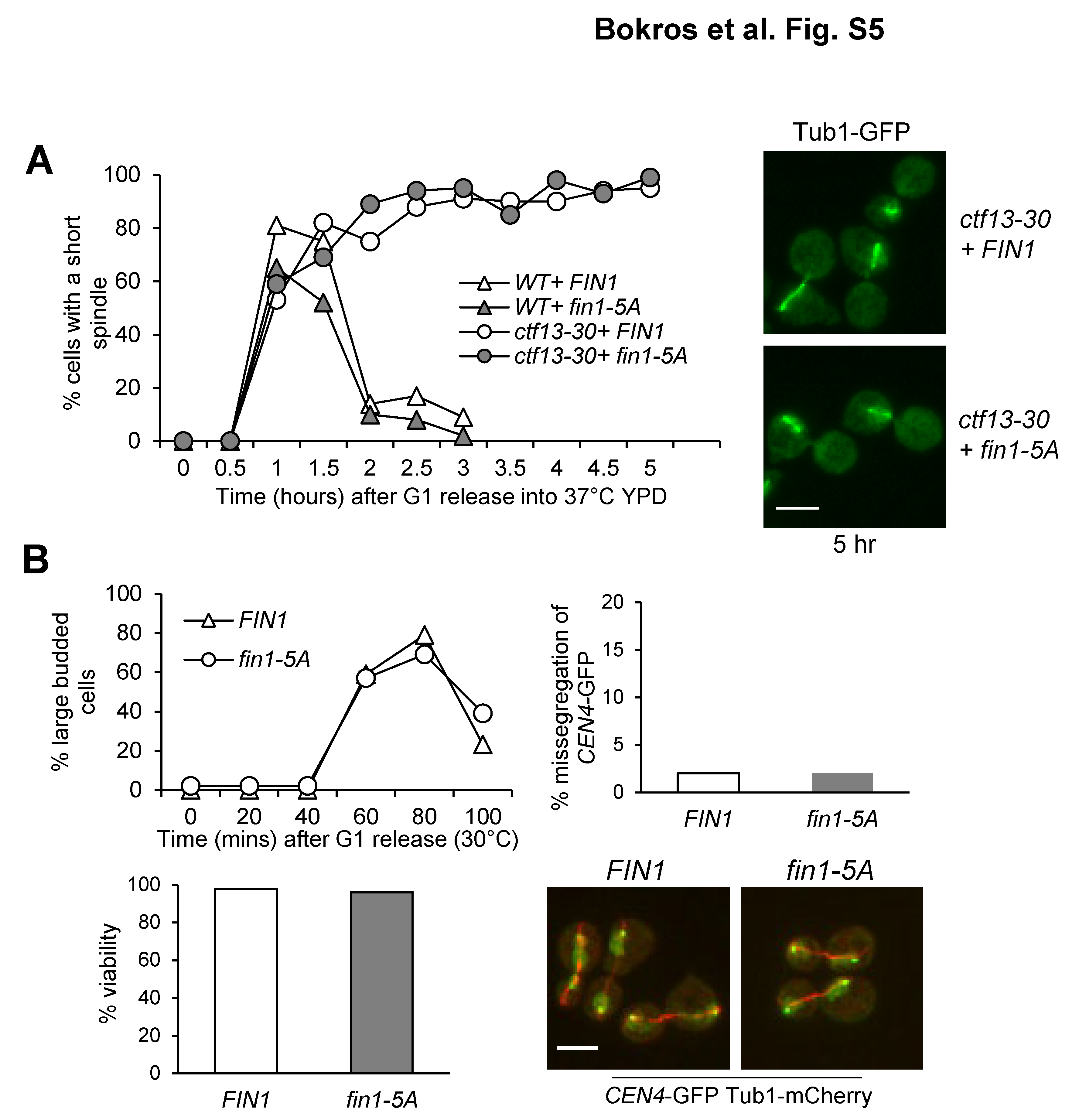

Supplement: S5 Fig — (A) ctf13-30 kinetochore mutant cells expressing Fin1-5A show metaphase arrest after long time incubation at non-permissive temperature. G1-arrested WT (4159-6-4) and ctf13-30 (4159-4-1) cells carrying FIN1 (pMB6) or fin1-5A (pMB7) plasmid and Tub1-GFP were released into 37°C YPD media for 5 hours. Cells were collected and fixed every 30 minutes to visualize spindle morphology (Tub1-GFP). The percentage of cells with short spindles was counted for each time point (n = 100). Spindles less than 3μM in length were counted as short spindles. Representative images were taken at 5 hours. Scale bar, 5μM. (B) Cells expressing Fin1-5A do not show increased chromosome missegregation. G1-arrested CEN4-GFP Tub1-mCherry cells (2167-17-2) carrying FIN1 (pMB6) or fin1-5A (pMB7) plasmid and were released into YPD media at 30°C. Cells were collected over time for budding index (top left). Cells at 80 minutes were plated onto YPD plates to examine plating efficiency (n ≥ 300) (Bottom left). We also collected cells at 80 minutes and fixed them to visualize spindle morphology (Tub1-mCherry) and CEN4-GFP segregation. The percentage of cells with CEN4-GFP missegregation is shown in the top right panel (n ≥ 100). Representative images are shown in the right bottom panel. Scale bar, 5μm. (TIF) [file pgen.1009592.s007.tif]

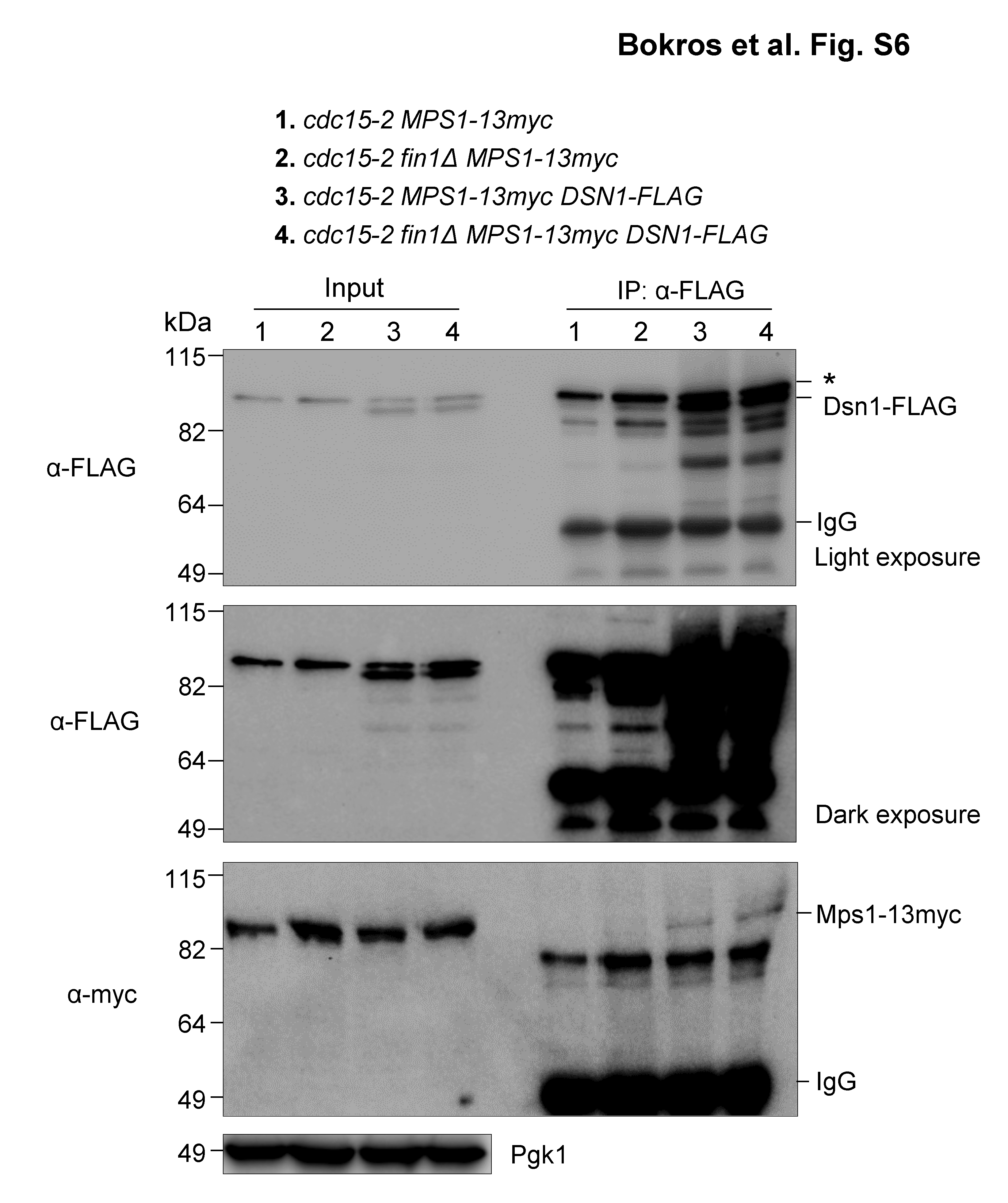

Supplement: S6 Fig — cdc15-2 MPS1-13myc (DS002), cdc15-2 fin1Δ MPS1-13myc (DS003), cdc15-2 MPS1-13myc DSN1-FLAG (DS004), and cdc15-2 fin1Δ MPS1-13myc DSN1-FLAG (DS005) cells were grown overnight in 15 mL YPD media at 25°C then switched to 37°C for 2 hours to inactive Cdc15-2. Cells were harvested and lysed via CryoMill, and Dsn1-FLAG protein was immunoprecipitated using M2 anti-FLAG beads. Dsn1-FLAG and Mps1-13myc were detected using anti-FLAG and anti-myc antibodies after separation on a 10% SDS-PAGE gel. Pgk1, loading control. * a non-specific band. (TIF) [file pgen.1009592.s008.tif]

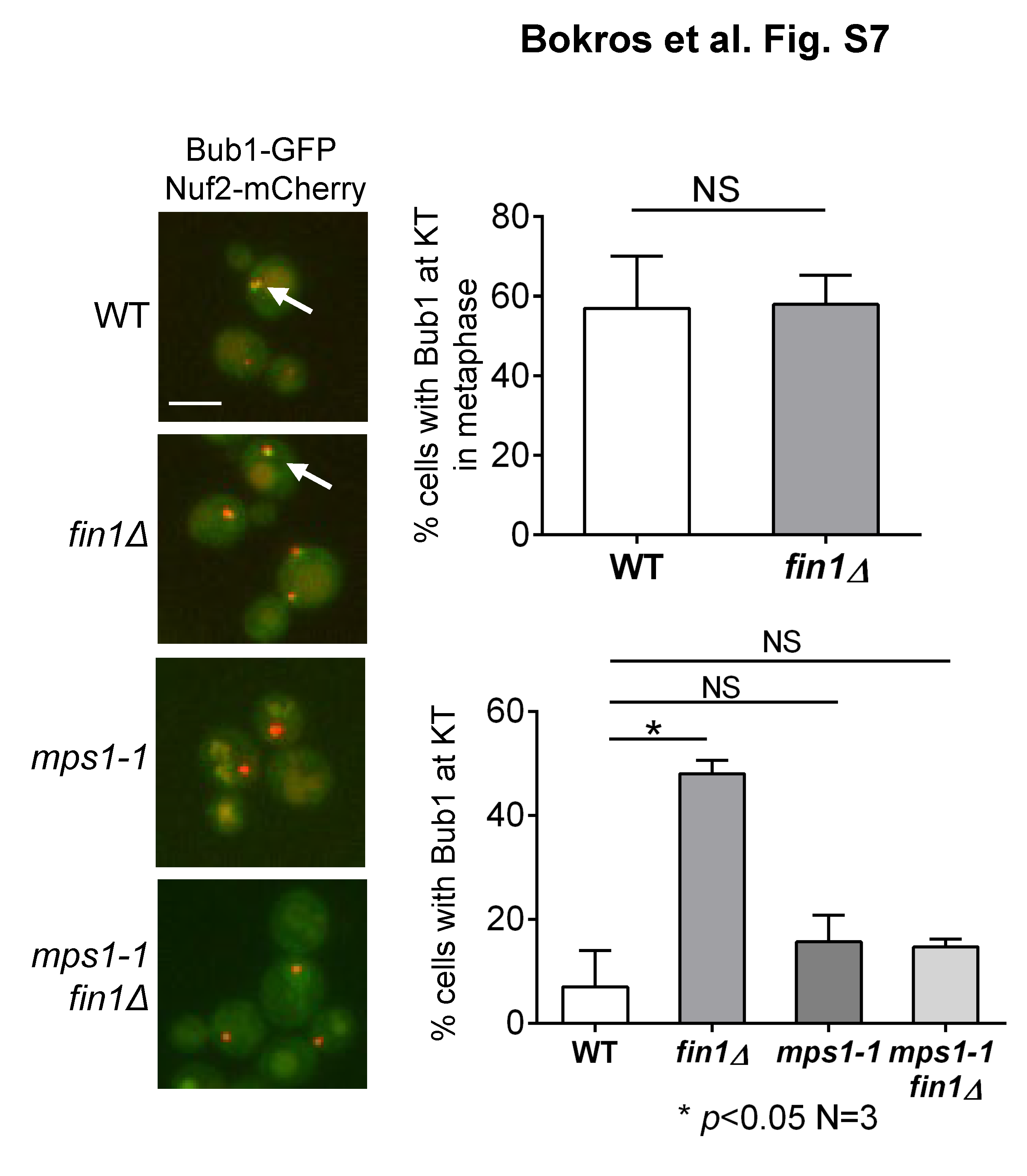

Supplement: S7 Fig — WT (3887-4-1), fin1Δ (3196-1-3), mps1-1 (4146-2-4), and mps1-1 fin1Δ (4171-3-1) cells containing Bub1-GFP and Nuf2-mCherry were grown to log phase at 25°C then switched to 37°C for 150 minutes. The frequency of co-localization of Bub1 and Nuf2 was counted in metaphase and anaphase cells (top right). WT and fin1Δ cell in anaphase were determined when the distance between two Nuf2 foci was greater than 3μm (n = 100), and the number is shown in the bottom right panel. No elongated spindles were observed in mps1-1 mutant cells after incubation at 37°C because of the function of Mps1 in spindle pole body duplication. The co-localization of Bub1-GFP with the single Nuf2 cluster was counted in mps1-1 mutant cells (n = 100), and the number is shown in the bottom right panel. The experiment was repeated three times and statistical significance of metaphase localization was determined by p < 0.05 using Wilcoxon rank sum test. Statistical significance of anaphase localization was determined by p < 0.05 using Kruskal-Wallis one-way ANOVA. Scale bar, 5 μm. (TIF) [file pgen.1009592.s009.tif]
