# Supplementary material for: Yeast Fin1-PP1 dephosphorylates an Ipl1 substrate, Ndc80, to remove Bub1-Bub3 checkpoint proteins from the kinetochore during anaphase
Source: PLoS Genet. 2021 May 25;17(5):e1009592. doi: 10.1371/journal.pgen.1009592 (PMC8184001; doi:10.1371/journal.pgen.1009592)
Supplement: Attachment — Submitted filename: 4-19-21-Response.docx [file pgen.1009592.s010.docx]

**Response for reviewer’s comments**

We appreciate the comments from each reviewer. We believe that the quality of this research work has been improved significantly by addressing all these comments. Below is the point-by-point response to these comments.

**Reviewer 1:**

*They used a fin1-5a mutant that prematurely associates with kinetochores (before anaphase and in nocodazole arrest) to show that its localization is not sufficient for displacing the Bub1-Bub3 complex. Several metrics were used to show that Fin1-5A at kinetochores does not inactivate the SAC, thought it was not ruled out that the SAC might be weakened. Longer time courses would be needed for that. It might be worth pointing this out in the text.*

**Response:** We agree that it is worth checking the efficiency of the SAC arrest in *fin1-5A* over a longer time period. We originally planned to repeat the experiment with nocodazole shown in Figure 3C over the course of 5 hours. However, our original nocodazole supplier stopped producing nocodazole and we were forced to order from another company. Unfortunately, this new nocodazole did not result in a strong metaphase arrest. We also borrowed nocodazole from neighboring labs but had similar shortcomings with the efficiency of the metaphase arrest. To test this possibility, we instead examined the metaphase arrest by visualizing spindle dynamics in temperature sensitive *ctf13-30* mutant cells, which arrest at metaphase due to impaired kinetochore function. We introduced the *FIN1* and *fin1-5A* plasmids into WT and *ctf13-30* mutant cells. We followed the spindle dynamics every 30 minutes after G1 release into 37°C for 5 hours and saw that *ctf13-30* carrying either or *fin1-5A* presented with short spindles, indicating metaphase arrest **(Fig. S5A).** We conclude that the SAC is not weakened by *fin1-5A* in cells with defective kinetochore-microtubule interaction even after long time incubation.

*After a nocodazole release, fin1-5A cells had reduced viability, but this should be done relative to plating untreated cells (no nocodazole). The experiments following CEN4 segregation are interesting, showing mis-segregation in fin1-5A cells after nocodazole release. If untreated fin-5A cells have reduced viability, then it might be worth following CEN4 segregation in untreated cells (I don’t think this is a necessary experiment for this manuscript, but it would be interesting to follow up).*

**Response:** We agree that repeating the same experiment without nocodazole treatment acts as a good control for the viability loss we see in *fin1-5A* in response to nocodazole. Using the same strains, we examined both viability loss and chromosome missegregation in *fin1-5A* cells without nocodazole treatment. The result shows that under normal conditions, no viability loss or chromosome missegregation was observed in cells expressing either *FIN1* or *fin1-5A* **(Fig. S5B).**

*Very interestingly, they present evidence that Bub1 kinetochore localization in anaphase depends on the CPC. The CPC should be re-localized to the spindle midzone in these anaphase cells, so this suggests CPC is staying at kinetochores. This would be a very valuable follow up study (but again, not necessary for this manuscript).*

**Response**: Our result shows that anaphase Bub1/Bub3 kinetochore localization is dependent on Ipl1 in *fin1Δ* cells. We speculate that this regulation is through the Ipl1-dependent phosphorylation of Ndc80 that occurs **before** anaphase entry (page 11). We agree that it will be interesting to examine CPC localization in *fin1Δ* cells. The regulation of CPC translocation by Fin1 will be the focus of our next story.

*They speculate that Mad1 dissociation from kinetochores occurs through a different mechanism, since Mad1 is not at the Bub1-Bub3 positive kinetochores in fin1 null telophase cells. This is interesting. It would be valuable to look in anaphase cells to be sure this result holds true (presently the experiment was done in the telophase arrested cdc15-2 background). This would be best investigated in a follow up study. There is already a lot of data in the current manuscript, and although it will be very interesting to follow up the Mad1 experiments, this is likely to open a whole lot of new questions.*

**Response:** As suggested, we examined Mad1 kinetochore localization in WT and *fin1Δ* anaphase cells (without *cdc15-2*). Similar to *cdc15-2* arrested cells, no Mad1 is colocalized with the kinetochore signal Nuf2-mCherry regardless of the presence of Fin1 **(Fig. S2)**.

**Reviewer 2:**

*1.Spc105 appears to be the major binding site for PP1 at KT (Rosenberg 2011), where Glc7/PP1 can then remove Mps1 phosphorylation at MELT motifs which are responsible for recruiting Bub3/Bub1. It is possible that the ipl1 (as well as ndc80-7A) and fin1 associated phenotypes are mediated through Mps1, so the authors need to analyze Mps1 localization. To address whether Mps1 is still at the KT and contributing to persistent Bub3/Bub1 anaphase localization, the authors should perform ChIP or kinetochore IPs (or any technique that will work to detect Mps1) from anaphase lysates in WT and fin1 mutants. Either result from such an IP (no change in Mps1 loading or Fin1 dependent change) would greatly strengthen (no change) or weaken (Fin1 dependent change) their model and thus should be addressed. Alternatively, the authors could use mps1 ts mutants to test if the Bub3/Bub1 anaphase localization in fin1 cells is independent of Mps1.*

**Response:** We agree that Mps1-KT localization could be the mechanism by which Fin1-PP1 regulates Bub1-Bub3 kinetochore localization. To test whether Mps1-KT localization in anaphase is the cause of persistent Bub1/Bub3 kinetochore localization in *fin1Δ* cells, we first attempted to visualize Mps1-GFP co-localization with the kinetochore. However, after numerous attempts at constructing a new *MPS1-GFP* strain, and also receiving a *MPS1-GFP* strain from a lab in our department (Dr. Robert Tomko), we were unable to visualize Mps1 localization at the kinetochore. We also performed co-IP assay using strains containing Mps1-13myc and Dsn1-FLAG following the protocol from Biggins’s lab. Because Bub1/Bub3 show strong kinetochore localization in *cdc15-2 fin1Δ* cells, we performed co-IP assay in cells arrested with *cdc15-2*. However, no increased interaction of Mps1 with kinetochore protein Dsn1 was observed in *cdc15-2 fin1Δ* compared to *cdc15-2* cells. Therefore, Fin1 unlikely regulates kinetochore localization of Mps1 (**Fig. S6**).

As suggested, we also used *mps1-1* mutant cells to test if Bub1 anaphase kinetochore localization in *fin1Δ* cells is dependent on Mps1 activity. We confirmed an increase of Bub1-KT localization in *fin1Δ* compared to WT*,* however in *mps1-1 fin1Δ* cells, this localization was greatly decreased **(Fig. S7)**. It is worth noting that fully separated Nuf2 kinetochore clusters were not observed in *mps1-1* mutants, and instead arrest as a signal Nuf2 cluster because of the function of Mps1 in SPB duplication (Winey, et al. 2009, JCB). These results suggest that the anaphase kinetochore localization of Bub1 in *fin1Δ* cells is in fact dependent on Mps1 activity.

*2.The claim that Ipl1 regulates Bub1/3 localization in anaphase is fine (but a bit odd considering Ipl1 is not on anaphase kinetochores), but the authors do not address the possibility that the ipl1 phenotype is due to prematurely localized PP1 which then leads to loss of Bub1/3 localization.*

**Response:** Our result shows that anaphase Bub1/Bub3 kinetochore localization is dependent on Ipl1 in *fin1Δ* cells. As discussed above, we speculate that this regulation is through the Ipl1-dependent phosphorylation of Ndc80 that occurs **before** anaphase entry, and prior to CPC de-localization from the kinetochore. This phosphorylation is reversed by Fin1-PP1 during anaphase for Bub1/Bub3 delocalization. Although we cannot exclude the possibility that Ipl1 acts upstream of Fin1-PP1to regulate Bub1-Bub3 KT localization, previous evidence shows that the kinetochore localization of Fin1-PP1 is regulated by Cdk1-dependent phosphorylation. Moreover, *ipl1* and *sli15* mutants largely abolished the kinetochore localization of Bub1/3 in *fin1Δ* mutants **(Fig. 4)**. Therefore, it is likely that Fin1-PP1 delocalizes Bub1/3 from the kinetochore by reversing Ipl1-imposed phosphorylation in anaphase. We have clarified this in the text (page 11).

*3.In multiple cases, the authors conclude that the SAC is compromised when all they do is look at budding or viability, assays that are not direct measures of the SAC. They make large conclusions that are not directly supported by the data. These conclusions need to be supported by experimental data or greatly toned down. Examples:*

*Fig 2c. They add nocodazole to bub mutants, but these mutants are not going to arrest so they don’t know what stage of cell cycle they are in yet make localization conclusions.*

**Response:** We agree! In the revised version, we modified the conclusions about SAC regulation.

In Figure 2C/2D, we performed experiments in *bub* mutant cells treated with nocodazole. We agree that these SAC mutant cells do not arrest at metaphase, and the conclusion was re-worded in the revised version. To address the interdependency of Bub1 and Bub3 on metaphase localization a different way, we performed an experiment looking at KT localization of Bub1/Bub3 in their respective mutants in asynchronous cells (no nocodazole arrest) **(Fig. S3)**. We determined metaphase as large-budded cells with two Nuf2 clusters less than 3μM apart. The result was consistent and showed that the metaphase localization of both Bub1 and Bub3 is greatly reduced in their respective mutants even without nocodazole treatment.

*Fig 3A. One can't conclude SAC might be competent based on a budding assay. A Pds1 time course is required to make this conclusion. However, this point is semi-irrelevant since this experiment was already published in 2009 paper (Genes and Dev) from Biggins lab.*

**Response:**  Yes, we agree. We showed the competency of the SAC in *FIN1* and *fin1-5A* after nocodazole treatment by examining Pds1 levels during cell cycle in **Figure 3C**. The result showed stabilized Pds1 level in *fin1-5A* cells in the presence of nocodazole.

*Fig 3D. They have no evidence that chromosome missegregation has anything to do with premature SAC silencing…Bub1 is important for biorientation so it is much more likely related to that than the SAC. It is confusing that the authors never mention the function of Bub1 in biorientation in this manuscript.*

**Response:** This part is rephrased in the revised version. We agree that we do not have evidence to support this conclusion. In the revised version, we speculate that the increase in chromosome missegregation in *fin1-5A* after nocodazole washout could be due to increased biorientation defect and/or compromised SAC. Page 9.

*Fig 7. There is nothing in the figure about the SAC, it is all viability which could be due to some other function.*

**Response:** We have clarified in the text that compromised SAC is only one explanation and we cannot exclude other possibilities.

*4.Gillet et al. (JCB 2004) previously addressed interdependency of checkpoint proteins for KT localization, this paper was not properly cited for these findings. Using ChIP, they found that Bub1 is dispensable for Bub3 binding at the centromere, which is in conflict with your microscopy findings. Please address this discrepancy experimentally (performing ChIP in your anaphase extracts to allay concerns) or textually.*

**Response:** We recognize that this conflicts with the ChIP result shown in Gillet et al. 2004, and this is mentioned in the text. Page 8

*5.The ndc80-7A mutant only shows a moderate decrease in Bub3/Bub1 anaphase localization compared to WT cells. The authors rightly conclude that this indicates Fin1 may be targeting other residues controlling Bub3/Bub1 localization. The authors should also textually include the complementary interpretation, which is that Ipl1 is targeting residues beyond the ndc80-7A mutant*.

**Response:** We agree. This section is reworded in the revised version.

***Minor points:***

*1) The manuscript should be explicitly clear as to where the ndc80-7A mutant comes from or what the residues are, as those unfamiliar with Ndc80 phosphorylation biology might not know what residues the authors are referring to*.

**Response:** We have clarified the Ndc80 mutant residues in the text. Page 12.

*3) Is it possible that the increased persistence of Bub3/Bub1 in fin1 anaphase is due to the increased amount of Bub3/Bub1 at the KT from a metaphase localization defect as seen in authors’ previous work (Bokros 2016 Cell Report)?*

**Response:** Using live-cell imaging shown in **Figure 1** and **Figure S1**, we showed that the KT localization of Bub1-Bub3 in *fin1Δ*  anaphase cells is dynamic, with frames alternating between localization and delocalization. Therefore, we speculate that the increase in Bub1/Bub3 KT localization is not just persistence from the canonical localization of these proteins in metaphase.

*4) Is there any in vivo evidence for Ipl1 phosphorylation of Ndc80 in anaphase?*

**Response:** Our explanation is that the Ipl1-phosphorylation of Ndc80 occurs prior to anaphase, however this phosphorylation is reversed during anaphase. This issue is clarified in the revised version. Page 11.

*5) Why is there a temperature sensitive effect on KT localization of Bub3/Bub1 in the fin1^ cells? Compare figure 4A/B to 1A.*

**Response:** In **Figure 1A**, the strains used are in *cdc15-2* background. In *cdc15-2* arrested cells lacking Fin1, high percentage cells show kinetochore localized Bub1/3. The strains used in **Figure 4** contain WT *CDC15*, because *ipl1* and *sli15* temperature sensitive mutants are used for this experiment. We speculate that Cdc15 activity may also play a role in Bub1/3 kinetochore localization. The combination of *FIN1* deletion and Cdc15 inactivation likely results in more dramatic KT localization of Bub1-Bub3. Page 10.

*6) The title is vague/non-specific and too broad*

**Response:** The new title is “Yeast Fin1-PP1 dephosphorylates an Ipl1 substrate, Ndc80, to remove Bub1-Bub3 checkpoint proteins from the kinetochore during anaphase”

**Reviewer 3:**

*All of the experiments are simple, straightforward and well presented. I have the following concerns about the data:*

*Figure 2.*

*Bub3 localization should be done in live cell imaging similar to Bub1 in Figure 1.*

*The interdependence of Bub1 and Bub3 for kinetochore localization is surprising. Does the failure to localize depend on Fin1? This should be tested.*

**Response:**  We agree that Bub3 localization using live-cell imaging in WT and *fin1Δ* cells is important to support our conclusion. Using the same methodology, we examined Bub3-KT localization in WT and *fin1Δ* cells during anaphase*.* Consistent with the Bub1 result, we found that *fin1Δ* cells also showed increase in Bub3-KT localization during anaphase compared to WT cells **(Fig. S1).** Page 6.

The anaphase KT-localization of Bub1 and Bub3 in *fin1Δ* cells is interdependent (**Fig. 2A/B)**. The KT-localization of Bub1/Bub3 is also interdependent in cells treated with nocodazole **(Fig. 2C/D)** and in asynchronous cells without nocodazole treatment **(Fig. S3A/B)**.

*Figure 3*

*SAC is active in Fin1-5A mutant with tagged Pds1 but tagged chromosomes missegregate.
The authors need an independent assay for chromosome missegregation in fin1-5A cells*

**Response:** We speculate that the increase in chromosome missegregation in *fin1-*5*A* after nocodazole washout **(Fig. 3D)** could be due to a few things. First, the increased chance of syntelic attachments during recovery, or because of premature Bub1 delocalization resulting in a biorientation defect and compromised SAC. For an independent assay, we have examined chromosome missegregation in untreated *fin1-5A* cells, but no increased chromosome missegregation was noticed compared to WT cells (**Fig. S5B**).

*Figure 5*

*The data are hard to normalize as described because it is not clear what the "main band" is. The better normalization would be to Pgk1. Set the ratio of the hyperphosphorylated band to Pgk1 band at t=0 to 1.0*

**Response:** We have adjusted the phosphorylation ratio to be normalized to Pgk1 **(Fig. 5B).**

*Figure 6*

*The ratio of Bub1-GFP and Bub3-GFP relative to Nuf2 M-cherry should be quantified in addition to the number of GFP positive cells.*

**Response:** Thanks for the suggestion. We tried to measure this ratio, but the high level of Bub3-GFP signal in the nucleus made it difficult for this measurement. However, we measured the ratio of Bub1/Bub3-GFP intensity at the kinetochore relative to the intensity at other nuclear regions. Total 20 cells were measured for each strain, and the results show increased kinetochore enrichment of Bub1/3-GFP in *fin1Δ* cells arrested with *cdc15-2*. Moreover, the kinetochore enrichment of Bub1 and Bub3 is interdependent. The new result is included in the revised version (**Fig. 2A and B**).

***minor*** ***concerns:***

*The strain list needs some corrections. I assume that supplemental table 1 has the relevant genotypes and not the complete genotypes. If that is the case, then indicate the background of all strains (W303a, S288C etc). The mating type locus should be uppercase (MAT) and the allele (a or alpha) should be bold.*

**Response:** Thank you for these suggestions. Only the relevant genotypes are shown in the strain list, and we have included this information in the revised version.

*The authors need to make clear that the experiments described at the bottom of page 8 are using plasmids in strains deleted for FIN1*

**Response:** We have updated the text accordingly. Page 9.

*There are several places where the writing is very "tentative" and the authors should be more conclusive.
For example:*

*"but untimely kinetochore localization of Fin1-5A may cause SAC silencing" is tentative and should be "but untimely kinetochore localization of Fin1-5A causes SAC silencing".*

*"Fin1-PP1 may antagonize Ipl1 to regulate Bub1 localization at the kinetochore" is tentative and should be "We determined if Fin1-PP1 antagonizes Ipl1 to regulate Bub1 localization at the kinetochore".*

*"the data suggest that Fin1-PP1 likely antagonizes Ipl1 activity" is tentative and should be "the data suggest that Fin1-PP1 antagonizes Ipl1 activity".*

*Tentative phrases throughout the manuscript should be edited.*

**Response:** We have taken this into consideration and removed tentative phrases throughout the text.
